# Determinants of incomplete immunization and factors for missed opportunities in urban Dhaka: A cross-sectional study

**DOI:** 10.1371/journal.pone.0326116

**Published:** 2025-06-17

**Authors:** Zahid Hasan Khan, Shamim Ahmed, Mohammad Ashraful Amin, Md Taufiqul Islam, Faisal Ahmmed, Motaher Hossain, Muhammad Shariful Islam, Tajul Islam A. Bari, Taufiqur Rahman Bhuiyan, Firdausi Qadri, Ashraful I. Khan

**Affiliations:** 1 Infectious Diseases Division, International Centre for Diarrhoeal Disease Research, Bangladesh (icddr,b), Dhaka, Bangladesh; 2 Maternal and Child Health Division, International Centre for Diarrhoeal Disease Research, Bangladesh (icddr,b), Dhaka, Bangladesh; 3 Integrated Management of Childhood Illness, Directorate General of Health Services, Mohakhali, Dhaka, Bangladesh; National Institute of Science Communication and Policy Research (CSIR-NIScPR), INDIA

## Abstract

Most vaccinations in the immunization schedule need two or more doses to elicit a protective immune response. Therefore, completion of all doses is crucial for achieving the best possible immunity. The objective of this study was to investigate the factors influencing missed opportunities of polio vaccination in children between the ages of 1–3 years in urban Dhaka. In 2018, according to the immunization card records or histories from parents/guardians, we sorted1–3-year-old children from areas of Dhaka South City Corporation who were not fully immunized. Immunization records were obtained from the Expanded Program on Immunization (EPI) card or maternal recall. Reasons for non-vaccination were documented. A total of 501 children were tracked down to determine the causes of their incomplete polio doses. Determinants of incomplete immunization and factors for missed opportunities were assessed by using bivariate and multivariable logistic regression model. The households with a child who had not received all the recommended vaccines had a considerably lower monthly income (18,000 BDT; p < 0.001). In both the complete and partial vaccination groups, the average family size was five people, and the average child age was 28 months. Education level of the household head after adjustment (AOR), the odds of the event occurring decrease by 25% with primary education (95% CI: 0.66, 0.85), p-value: < 0.001). Occupation of the household head for rickshaw/van/cart puller, AOR, the odds increase even more, with the event being 3.15 times more likely for this occupation (95% CI: 1.95, 5.08) and statistical significance (p-value < 0.05). Again, for daily wager AOR, 2.16 times higher for daily wagers (95% CI: 1.35, 3.45) and statistical significance (p-value: 0.001). This study identifies sociodemographic factors that influence incomplete childhood immunization in this urban area of Dhaka. In order to improve the coverage, the identified factors need to be mitigated and policymakers should focus on enhancing community engagement, combating misinformation and increasing the accessibility of vaccination services.

## Introduction

Immunization involves administering vaccines to achieve immunity against infectious agents and prevent related diseases [1]. Childhood immunization is the safest method to protect against life-threatening diseases like polio and is recognized as the most effective and economical intervention to reduce childhood mortality and morbidity [2,3]. In 2018, an estimated 700,000 children under five died from vaccine-preventable diseases, mostly in low- and middle-income countries [4,5]. To meet the Sustainable Development Goals (SDGs) by 2030, all countries must reduce newborn deaths to at least 12 per 1,000 live births and under-5 mortality to at least 25 per 1,000 live births. The World Health Organization (WHO)‘s Immunization Agenda 2030 aims to ensure all children receive recommended vaccines regardless of location, age, socioeconomic status, or gender [6]. The Expanded Program on Immunization (EPI), established in 1974 with WHO support [3,7] provides basic vaccines globally. Immunization currently prevents 2–3 million deaths annually [8], with an additional 1.5 million deaths preventable by expanding global vaccination coverage. Bangladesh’s Expanded Programme on Immunization (EPI) adheres to the vaccine schedule recommended by the WHO is BCG (Bacillus Calmette–Guérin) at birth, Penta (diphtheria-tetanus-pertussis-hepatitis B-Haemophilus influenza type b) and OPV (oral polio vaccine), two doses of fIPV (fractional-dose inactivated polio vaccine) at 6 and 14 weeks of age. PCV (pneumococcal conjugate vaccine) in three doses at 6, 10, and 14 weeks of age, followed by measles vaccine (MR) at 9 and 15 months [9]. Despite progress, many children, especially in low- and middle-income countries, remain unvaccinated [7].

However, the World Health Organization (WHO) and the United Nations Children’s Fund (UNICEF) predict that 1.5 million children suffer each year from vaccine-preventable diseases due to low vaccination coverage [10]. Even though childhood immunizations are one of the most effective disease prevention strategies globally, vaccine hesitancy has always influenced uptake and effectiveness [11–13]. Reasons related to religion and culture [14–16] challenges, and opinions of parents/guardians who reject or partially accept childhood immunizations, such as vaccine safety and lengthy vaccine repercussions, have been reported [17–20]. Some of the parents have strong belief that, children gets too many vaccines at once, can’t prevent them from getting infected or feels that it will hamper natural growth. [21,22]. Compared to other South Asian countries, the vaccination coverage for children is the highest in Bangladesh [23]. Though, it is found that full immunization coverage among children aged 12–35 months was 86.17% in 2011, 85.13% in 2014, and 89.23% in 2017–18 [24]. Childhood vaccination prevents roughly 200,000 deaths in Bangladesh each year despite the noteworthy success. Bangladesh is on the list of the top ten countries with the highest childhood mortality globally [7,25]. The vaccinated person’s immune response depends on the type of vaccine used, the number of doses given, and if the person has already been vaccinated against the disease. Bangladesh’s efforts to reduce child mortality have relied heavily on the EPI services. Therefore, to achieve the health-related SDG, especially the target of reducing preventable deaths of newborns and under-five children by 2030, it is crucial to expand the coverage of vaccination of children. More than 50 million deaths can be averted through childhood immunization [26]. Several studies have explored immunization coverage in Bangladesh, few have focused on the urban context particularly the determinants of incomplete immunization and missed opportunities among children in densely populated urban areas like Dhaka. Urban-specific barriers such as high population mobility, informal settlements, and health system fragmentation are often underrepresented in the literature. Our study addresses this gap by investigating these unique challenges in an urban setting, thereby contributing evidence to guide targeted interventions. So, the objective of this study was to investigate the factors influencing missed opportunities of polio vaccination in children between the ages of 1–3 years in urban Dhaka. Understanding the key factors influencing childhood vaccination is crucial for developing strategies to enhance vaccination coverage and reduce child mortality and morbidity.

## Materials and methods

### Ethics Statement

The protocol was approved by the Research Review Committee (RRC) and Ethical Review Committee (ERC) of the International Centre for Diarrhoeal Disease Research, Bangladesh (icddr,b). Informed written consent was obtained from the parents or legal guardians of the children. However, we did a secondary analysis of the data.

### Study site

The study was conducted in the southern city corporation urban slum areas of Dhaka city, Bangladesh, which included wards 14, 22, 34, 55, 56, 57 in Rayerbazar, Hazaribagh, and Kamrangirchar. These areas were densely populated and characterized by urban slums and considerable infrastructure is available to support the study. The study area has an aggregate population of approximately 300,000 persons, where there is a high population density (3.4 persons per room). Access to piped water is high (96% of households) but the quality of water is suspect due to breakdown of underground pipes, and to frequent lapses in water pressure leading to contamination of piped water by nearby sewage pipes, which also are ridden with leaks. About 81% of households have access to improved sanitation, but dense crowding makes proper management of solid waste very difficult. As well, the population has demonstrably low levels of knowledge about proper personal and food hygiene, and makes little personal investment in such hygiene. So, represent densely populated urban settings with a high concentration of low-income households, informal settlements, and variable access to healthcare services factors known to influence immunization coverage.

### Study design and data collection

The original study was conducted following a randomized controlled trial that was conducted among healthy children aged 1–3 years who had previously received no more than one dose of oral polio vaccine (OPV) and had not received any doses of inactivated polio vaccine (IPV) or oral cholera vaccine prior to enrollment. The original sample size was calculated based on the hypothesis that the immune response to bOPV and Oral Cholera Vaccine administered simultaneously is non-inferior to the immune response when administered individually. Specifically, for poliovirus types 1 and 3, we assumed a 50% seroconversion rate, a non-inferiority margin of 15%, a significance level of 5%, and a study power of 85%. Based on these parameters, we estimated that 173 participants would be needed in each of the three groups. To account for a 10% attrition rate, the total enrollment target was set at 579 children who had received either no or one dose of bOPV [27]. However, for this secondary analysis, which focuses on immunization coverage and determinants, we included only participants with complete and verified data. Due to loss to follow-up and missing outcome variables, the final analytic sample comprised 501 children. Eligible children were enrolled after obtaining written informed consent from their caretakers followed them for one year of enrolment. The eligible children were invited to the study field office and enrolment was started from May 11, 2018 and follow-up completed on 27 August, 2019. During the study, we collected the immunization records from the participants either from the EPI cards or history from the parents/guardians. We prioritized verification through the child’s EPI card whenever available. In cases where the EPI card was not present, maternal recall was used with careful probing and cross-checking against the standard vaccination schedule to improve accuracy. Reasons for non-vaccination were also documented. The questionnaire was adapted from standardized tools previously used in immunization coverage surveys in Bangladesh and was reviewed by public health experts for content validity. Prior to the main data collection, the tool was revised accordingly. Additionally, all interviewers received intensive training on the questionnaire content, interview techniques, and ethical considerations to ensure consistency and minimize interviewer bias.

### Operational definitions used in this analysis

In Bangladesh, the standard polio virus vaccination schedule includes bivalent OPV (bOPV) at birth and at 6, 10, and 14 weeks of age, with IPV (containing all three serotypes) administered intramuscularly at 14 weeks of age. We used the following definitions for this analysis.

#### Complete vaccination:

A child who received the following vaccines within 15 months of age. One dose of Bacillus Calmette-Guérin (BCG), three doses each of the DPT-HepB-Hib (pentavalent) vaccine, three doses of OPV, two doses of IPV, three doses of pneumococcal conjugate vaccine (PCV), and two doses of the measles-rubella vaccine.

#### Incomplete vaccination:

A child aged 1–3 years who missed at least one dose of the OPV vaccines was considered incompletely vaccinated in this study. There is no known cure for polio; vaccination, primarily with live-attenuated OPVs, remains the single, most effective preventive and control strategy. We used Oral Polio Vaccine (OPV) status as the primary indicator due to its multi-dose requirement and the observed tendency for dropouts between successive doses in previous studies. OPV coverage serves as a sensitive marker for identifying missed opportunities and service delivery gaps.

### Statistical analysis

Descriptive statistics were used to summarize the demographic and socio-economic characteristics of the study participants. Frequencies and percentages were calculated for categorical variables, while means and standard deviations were computed for continuous variables. Significance of the associations and the difference between groups were measured using chi-square tests for categorical variables and t-tests for continuous variables. To identify determinants of incomplete vaccination, bivariate analyses were initially conducted using logistic regression model. Variables with a p-value < 0.20 in the bivariate analysis were included in a multivariable logistic regression model to control for potential confounders. Crude odds ratios (COR) and adjusted odds ratios (AOR) with 95% confidence intervals (CIs) were measured by exponentiating the coefficients of the model and reported to assess the strength of associations. The reasons for zero dose and one dose of vaccination were also explored with number and percentage and compared by using Fisher exact test with Monte Carlo simulation to compute the p value for the large table. R version 4.4.2 was used for all the statistical analysis and MS Excel 2019 was used to generate the figures. We conducted a complete case analysis, excluding observations with missing values for key variables from the final regression models. The proportion of missing data was low and not systematically related to the outcome or exposure variables, minimizing the risk of bias. The child’s age, sex, household education, household income, and occupation. variables were selected based on prior literature and their theoretical and empirical associations with immunization status. Including these covariates allowed us to adjust for potential confounding and isolate the independent effects of key predictors.

## Results

**Fig 1:**
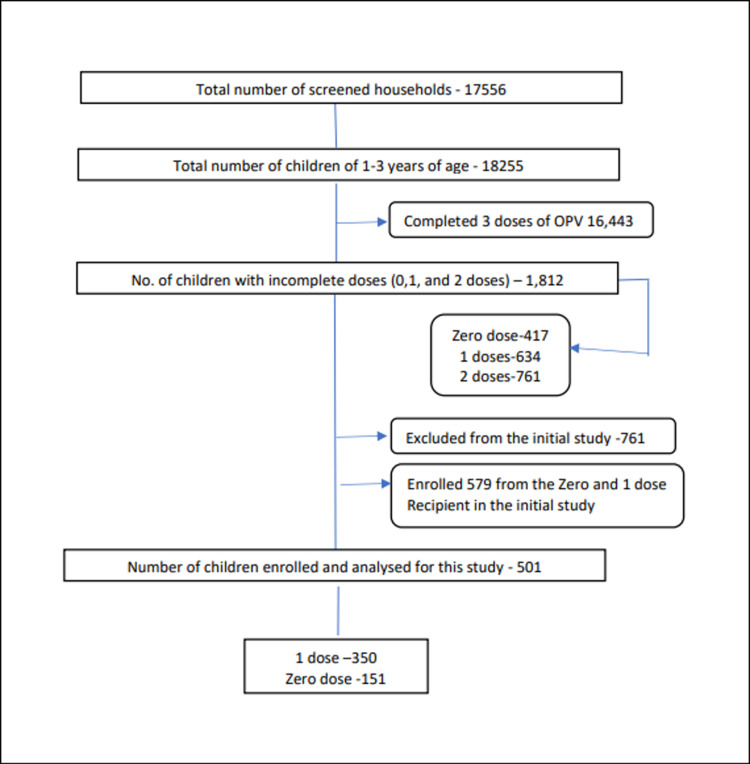
CONSORT of participants with OPV status.

A total of 17,556 households were visited, and 18, 255 children of 1–3 years were found on screening for the study. Of 18, 255 children 15, 814 received 3 doses of OPV, 761 had 2 doses, 634 had 1 dose, and 417 received no dose of OPV. Finally, 501 children out of 579 study participants were followed up to find out why they missed the dose or even received no dose of OPV (Fig 1).

Parents or legal guardians visited our study clinic with their children three times over two months. Each visit took about 60 minutes. During the visit, study staff asked questions about the child’s health, examined the child, collected blood samples (2/3 ml), administered respective vaccines, watched for any vaccine reactions, and scheduled the next visit. A total of 18,255 children aged 1–3 years were screened for the study, of which 16, 443 have completed EPI vaccination, and other 1, 812 have incomplete vaccination. The mean age of the household heads was ~ 34 years for both complete and incomplete vaccination groups. The monthly income of the household with incomplete-vaccination-child was significantly lower (18,292 BDT) than that of the complete vaccination group (p < 0.001). The average family size was ~ 5 members, and the child’s age was ~ 28 months in both complete and incomplete vaccination groups. Most household heads (~98%) were male irrespective of the complete/incomplete vaccination group. Secondary and above education was the highest level (49.6%) for most household heads, and the proportion was higher in the complete vaccination group (49.6%%) than in the incomplete group (29.5%%). Most household heads were driver/tailor/trader; 41% in the complete vaccination group and ~30% in the incomplete vaccination group, followed by service holders & others (~30% vs ~ 22%) (Table 1).

**Table 1:** Distribution of background characteristics of the study participants (N = 18255).

Variables	Vaccination status		*p-*value^*^
	Complete (N = 16443)	Incomplete (N = 1812)	
	Mean (SD)	Mean (SD)	
**Age of the household head**	33.87 (7.3)	34.34 (8.5)	0.023
**Monthly income (in BDT) of the household**	20798 (17671)	18292 (9867)	<0.001
**Number of family member**	4.53 (1.7)	4.71 (1.7)	<0.001
**Age of the child (in months)**	28.49 (10.0)	28.45 (9.8)	0.865
	***N *(%)**	***N *(%)**	
**Sex of the household head**			
Male	16106 (98.0)	1765 (97.4)	0.148
Female	337 (2.0)	47 (2.6)	
**The education level of the household head**			
No education	2989 (18.2)	616 (34.0)	<0.001
Primary	5306 (32.3)	662 (36.5)	
Secondary and above	8148 (49.6)	534 (29.5)	
**Occupation of the household head**			
Unemployed	351 (2.1)	33 (1.8)	<0.001
Rickshaw/van/cart puller	1267 (7.7)	334 (18.4)	
Daily wager	3111 (18.9)	510 (28.1)	
Driver/tailor/trader	6745 (41.0)	542 (29.9)	
Service holder and others	4969 (30.2)	393 (21.7)	
**Sex of the child**			
Male	8462 (51.5)	890 (49.1)	0.061
Female	7981 (48.5)	922 (50.9)	

* *p*-values were obtained from t-test for continuous variables and chi-square test for categorical variables.

### Risk measures of socio-demographic determinants

Monthly income is strongly associated with the outcome, with a 1% increase in income leading to a 1% increase in the odds of the event (AOR: 1 (95% CI: 1, 1), p-value: < 0.001). In case of number of family members; After adjusting for other variables, it was significant (95% CI: 1.06, 1.13), p-value: < 0.001). Age of the Child (in Months) does not significantly affect the outcome. Education level of the household head After adjustment, the odds of the event occurring decrease by 25% with primary education (95% CI: 0.66, 0.85), and statistical significance p-value: < 0.001). Secondary and above (AOR), after adjustment, the odds decrease by 53%, indicating that higher education has a strong protective effect (95% CI: 0.41, 0.54) and statistical significance (p-value: < 0.001). Occupation of the household head for rickshaw/van/cart puller, after adjustment, the odds increase even more, with the event being 3.15 times more likely for this occupation (AOR): 3.15 (95% CI: 1.95, 5.08) and statistical significance (p-value < 0.05). Again, for daily wager After adjustment, the odds increase to 2.16 times higher for daily wagers (95% CI: 1.35, 3.45). and statistical significance (p-value: 0.001) (Table 2).

**Table 2:** Determinants of incomplete vaccination.

Variables	COR (95% CI)	p value	AOR (95% CI)	p value
**Age of the household head**	1.01 (1, 1.01)	0.010	1 (0.99, 1.01)	0.629
**Monthly income (in BDT) of the household**	1 (1, 1)	<0.001	1 (1, 1)	<0.001
**Number of family member**	1.06 (1.03, 1.09)	<0.001	1.1 (1.06, 1.13)	<0.001
**Age of the child (in months)**	1 (0.99, 1)	0.867	–	–
**Sex of the household head**				
Male	Ref.		Ref.	
Female	1.27 (0.93, 1.73)	0.126	1.59 (1.07, 2.37)	0.021
**Education level of the household head**				
No education	Ref.		Ref.	
Primary	0.61 (0.54, 0.68)	<0.001	0.75 (0.66, 0.85)	<0.001
Secondary and above	0.32 (0.28, 0.36)	<0.001	0.47 (0.41, 0.54)	<0.001
**Occupation of the household head**				
Unemployed	Ref.		Ref.	
Rickshaw/van/cart puller	2.8 (1.92, 4.09)	<0.001	3.15 (1.95, 5.08)	<0.001
Daily wager	1.74 (1.21, 2.52)	0.003	2.16 (1.35, 3.45)	0.001
Driver/tailor/trader	0.85 (0.59, 1.23)	0.402	1.4 (0.88, 2.24)	0.158
Service holder and others	0.84 (0.58, 1.22)	0.362	1.38 (0.86, 2.22)	0.176
**Sex of the child**				
Male	Ref.		Ref.	
Female	1.1 (1, 1.21)	0.058	1.1 (1, 1.22)	0.054

**Fig 2:**
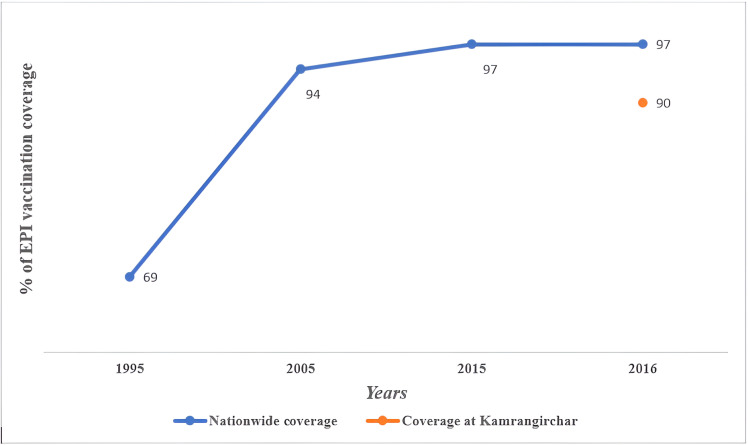
OPV coverage by nationwide and at study areas (as per enrollment period).

In 1995, only 69% of children nationwide had been vaccinated with 3 doses of OPV delivered through the EPI. After a decade, the coverage reached 94% and kept its upward trend to 97% at the end of next decade; even was steady until 2016. Whereas, the OPV coverage at study areas was only 90% (Fig 2) which is also below the nationwide coverage of 2016.

**Fig 3:**
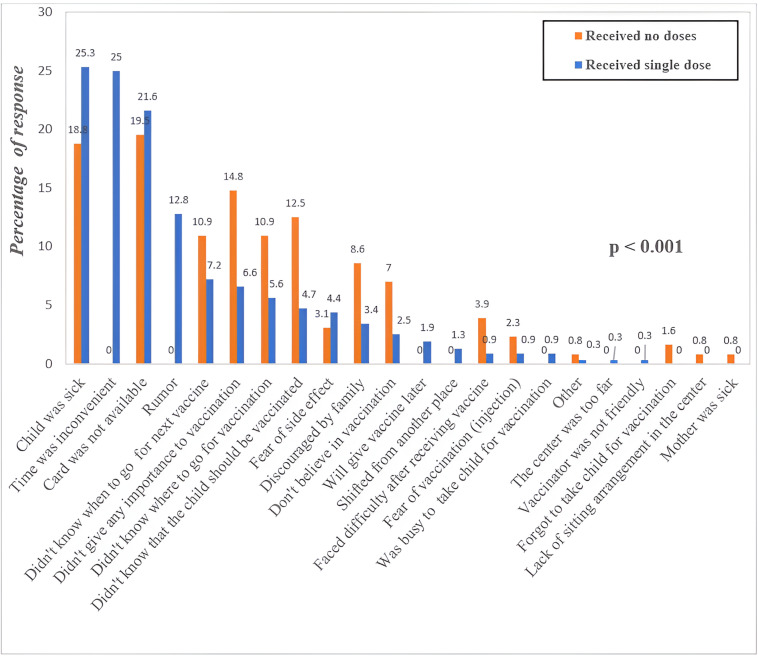
Reasons given by parents/guardians of incompletely vaccinated children (Zero-dose and one-dose OPV vaccination).

Among 1812 incomplete-vaccinated children, the proportion of females (51%) was slightly higher than males (49%). Various reasons were adduced by the parents/guardians for the incomplete OPV vaccination of their children. These include 25.3% child were sick, time was inconvenient (25%), unavailability of card (21.6%), rumor (12.8%), didn’t know when to go for the next vaccine (7.4%), reluctant to understand the importance of vaccination (6.6%), lack of information about vaccination site (5.6%), lack of importance to be vaccinated (4.7%), fear of side effects (4.4%), discouraged by family (3.4%); did not believe in vaccination, the decision to vaccinate later, shifted to another place, faced difficulties after receiving the vaccine, fear of vaccination, busy to take the child for vaccination, long distance to vaccination center, and vaccination was not friendly. Significant difference for the reasons between no vaccination and single dose of vaccination has found. Didn’t give any importance to the vaccination, didn’t know where to go for vaccination, didn’t know that child should be vaccinated, etc. were the higher reasons for no vaccination whereas, time was inconvenient, child was sick and rumor were high in proportion among single dose of vaccinated participants (Fig 3).

## Discussion

Immunization is a cornerstone of public health, significantly contributing to the reduction of childhood mortality rates. Our study sheds light on the factors influencing incomplete immunization in children aged 1–3 years in urban Dhaka, Bangladesh, particularly focusing on the oral polio vaccine (OPV). A major finding of our study is the correlation between household income and vaccination status. Households with a monthly income of less than 18,000 BDT were significantly more likely to have children with incomplete vaccinations. This finding is consistent with a study conducted in Nepal, where lower socioeconomic status was associated with reduced access to vaccinations [28]. Similarly, a study in Pakistan found that families with higher income levels were more likely to have fully vaccinated children, suggesting that economic constraints can impede timely access to healthcare services [29]. The economic burden on lower-income families may lead to prioritizing immediate needs over preventive healthcare measures, such as vaccinations. Moreover, our research identified that larger family size was associated with a higher likelihood of incomplete vaccinations, with families having more members being more likely to miss vaccinations. This observation resonates with findings from a study in rural Bangladesh, which reported that larger household sizes often lead to resource allocation challenges, thereby negatively impacting children’s health outcomes [30]. In larger families, the attention and resources for healthcare may become difficult which contributing to missed vaccination opportunities. The role of the household head’s education level emerged as another critical factor influencing vaccination status. Children whose household heads had attained primary or secondary education were less likely to be incompletely vaccinated compared to those with uneducated heads of households. This finding aligns with global evidence showing that maternal and paternal education significantly impacts child health behaviors, including vaccination uptake [31]. Educated parents are often more aware of the importance of immunization and may seek healthcare services more diligently, thereby ensuring timely vaccinations for their children.

Our study also found that children with female household heads had higher likelihood of receiving incomplete vaccinations. This finding echoes the results of a study in India, where children from female-headed households were found to have lower vaccination coverage due to societal and cultural barriers [32]. Female-headed households may face additional challenges in accessing healthcare services due to prevailing gender norms and limited autonomy in decision-making regarding children’s health. The qualitative data collected from parents regarding the reasons for incomplete OPV vaccination further illustrate the complexities of vaccination uptake. A notable parent reported that their child was sick at the time of vaccination, indicating a missed opportunity due to health concerns. This aligns with findings from a study in Nigeria, which found that parental perceptions of a child’s health significantly influenced vaccination decisions [33]. Additionally, logistical barriers such as inconvenience of time and unavailability of vaccination cards were commonly reported, suggesting that improving the accessibility and convenience of vaccination services could enhance uptake. The influence of misinformation and vaccine hesitancy also surfaced in our study. Vaccine hesitancy has been increasingly recognized as a barrier to immunization worldwide. A systematic review highlighted that misinformation about vaccines often spreads through community networks, influencing parents’ decisions and leading to decreased vaccination coverage [34]. Addressing these misconceptions through targeted community engagement and education could play a crucial role in improving vaccination rates. Furthermore, logistical challenges such as long distances to vaccination centers and busy schedules were highlighted as barriers. A study in Kenya reported similar findings, emphasizing the need for innovative solutions, such as mobile vaccination units, to reach underserved populations [35]. Improving access to vaccination services, especially in urban settings where transportation can be challenging, may reduce missed opportunities.

Research in Pakistan identified several key factors affecting whether children completed their immunizations, including where they were delivered, their gender, and their mother’s education level and region of residence [36]. Children were less likely to have incomplete immunizations if their mothers were educated or if they were delivered at a health facility. In addition, girls were more likely to be incompletely immunized compared to boys. Similarly, studies in India found that maternal education, socioeconomic status, gender, and place of delivery were significant predictors of incomplete immunization [37]. Furthermore, a 2016 study in Nepal showed that 78.2% of children were fully immunized, while 21% were under-immunized, and 0.8% were un-immunized. Possessing an immunization card was strongly linked to being fully immunized, and children whose mothers had education beyond secondary school or were employed had higher odds of being fully immunized [38].

This study has several limitations because most of the sample comprise children from low socio-economic from a single peri urban area capture factors related with the supply of vaccine for childhood immunization. However, a formal trend analysis using time-series methods (e.g., linear regression) was not feasible due to differences in data granularity and time intervals. The national immunization data are aggregated annually and may not align with the more detailed, sub-annual or campaign-specific data collected in the study area. Additionally, the study area experienced unique intervention timelines and local contextual factors that differ from national programmatic efforts, limiting the validity of direct statistical comparisons. Recall bias as vaccination status was partly self-reported by caregivers, there is a risk of over- or under-reporting, especially in the absence of vaccination cards. Generalizability; since the study was conducted in an urban setting, the findings may not be directly applicable to rural areas where healthcare access, infrastructure, and socio-cultural dynamics differ significantly. Selection bias; while we employed systematic recruitment procedures, individuals who participated may differ from those who did not, potentially influencing the representativeness of our sample.

We suggest that urban immunization programs can reduce missed opportunities by integrating vaccination services into routine outpatient and child health visits, improving provider training on checking vaccination status, and utilizing digital reminder systems. Additionally, we highlight the importance of targeted outreach programs such as mobile clinics and community health worker engagement in underserved urban areas to increase accessibility and address vaccine hesitancy.

## Conclusion

Our study revealed that sociodemographic factors, including household income, family size, education level of household heads and the gender of the household head significantly influenced the immunization status of children in urban Dhaka. These findings emphasized the necessity for tailored interventions that consider these needs for targeted interventions that address these determinants to improve vaccination coverage. Policymakers should focus on enhancing community engagement, combating misinformation, and increasing the accessibility of vaccination services. By implementing this so, we can contribute to achieve the global goal of reducing childhood mortality and morbidity associated with vaccine-preventable diseases.
